# Understanding the causes of mortality post-transplantation - there is more than meets the eye

**DOI:** 10.1590/2175-8239-JBN-2018-0002-0003

**Published:** 2018-06-25

**Authors:** Leonardo V. Riella

**Affiliations:** 1 Harvard Medical School, Brigham and Women's Hospital, Renal Division, Transplant Research Center, Boston, MA, USA.

**Figure 1:**
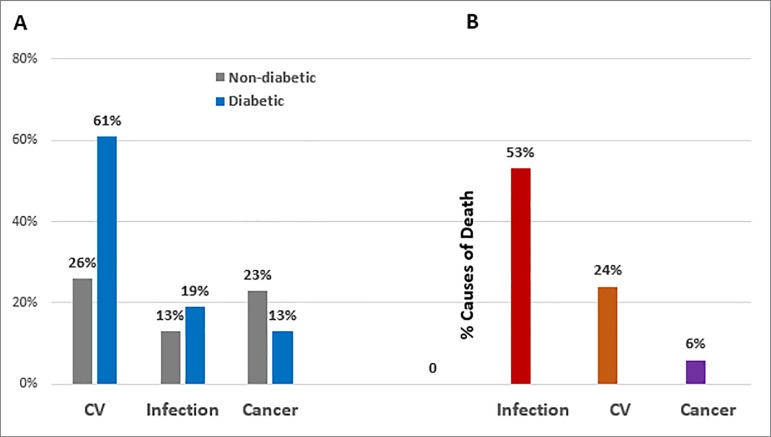
Causes of death post-transplantation. A, Causes of death in diabetic and non-diabetic recipients in a single center in the USA with mean follow-up of 4 years (adapted from Cosio et al. AJT 2008:593). B, Causes of death in recipients in a single center in Brazil with mean follow-up of 5 years (6).

Death with a functioning graft is the leading cause of late graft loss. The underlying cause of death post-transplantation varies in different populations. Therefore, it is an essential task of any individual transplant program to understand those causes in order to improve long-term outcomes. In the US, the three leading causes of death after transplantation are cardiovascular disease, malignancy, and infections. Cosio et al. reported that while cardiovascular mortality is higher in diabetics post-transplantation, cancer is the most common cause of death in non-diabetics (Figure 1A).^1^ On the contrary, infections seem to be a dominant cause of mortality in developing countries.^2^^-^ 5 In addition to traditional risk factors, such as prior history of heart disease, diabetes, and recipients' age, pre-transplantation length on dialysis, rejection events, and donor organ quality may all play additional roles in shaping the post-transplant course. Importantly, socioeconomic factors and environmental exposures further complicate and determine the ultimate outcome.

Tedesco's group reports a case-control study in which 1,873 kidney transplant recipients with at least 5 years of follow-up were screened.^6^ In total, 162 deaths were matched to 137 controls. The groups were matched according to date of transplant, recipient's age, donor's age, gender, type of donor, and induction with thymoglobulin. Longer time on dialysis and diabetes were more common in the case group, while no differences were seen in marital status, religion, and human development index between groups. Furthermore, patients with lower level of education and unemployed were more frequent in the case group. Delayed graft function was more frequent in patients that died and there was a trend towards higher number of rejection episodes and lower eGFR compared to controls. Potential limitations of this study include the single center and case-control design, which may hide important risk factors. Nonetheless, it is useful to hypothesize potential associations between mortality and certain recipients' risk factors.

Infection was the leading cause of death in 53% of patients with a greater proportion occurring in the first year post-transplantation (Figure 1B). Among these, pneumonia and sepsis infection accounted for the majority of infections linked to death. One of the crucial questions here is why such a high rate of infection is associated with mortality in this population? Since the choice of immunosuppression is not that different from other centers in the world and the infection mortality seems not associated with endemic infectious diseases of Brazil, what accounts for this difference? Are patients being diagnosed later and with infection, and therefore not having timely antibiotic treatments/fluid resuscitation and immunosuppression reduction during sepsis?

As a physician trained in Brazil and currently practicing in the United States, it is clear to me that a major difference in medical practice between the two countries has little to do with the physicians themselves but rather with the medical team including physicians, nurses, and physician assistants. Physicians and nurses are extremely well trained in the US and capable of monitoring patients closely enough to spot significant changes at a time in which interventions are most likely to be effective. I currently follow over 200 transplanted patients in my clinic and work with 2 nurse practitioners, who allow me to concentrate on the critical issues while delegating part of the care to the them. Furthermore, nurses act as gatekeepers and have the authority to question the physician's actions if deemed incorrect. Multiple students and residents from Brazil came as observers in my transplant service in the past few years and one of the most noticeable differences from practice in Brazil reported by them was how thoroughly the nurses knew their individual patients and how actively involved with care decisions they were. A healthcare system focused mostly on the physician is a utopic model since the doctor to patient ratio will always be too low. Certainly, the issue does not affect people with high socioeconomic status and the consequences are predominantly noticed by the socially disadvantaged population. As a suggested solution, rather than importing poorly trained physicians from other countries, Brazil should invest in training nurses and physician assistants to have more accountability in patient care, creating stronger teams and consequently improving patient outcomes. The physician should be the team leader, defining clear purposes, goals, and individual responsibilities of each team member.

However, I have to acknowledge that the opinion given above is just one potential hypothesis/action plan and most likely there are multiple factors contributing for the observed high rates of infectious deaths post-transplantation. Does poor nutrition, lack of effective medications due to institutional financial restrains or lack of patient education play a role? The current literature does not allow a clear understanding of all these variables but I am confident that the bright transplant physicians in Brazil may have additional ideas of the reasons for such a high mortality from infection. Fortunately, the dialogue is open and detailed work such as the one presented by Tedesco's group will inspire others to study their own patient population and suggest feasible plans to improve long-term transplant outcomes. Most importantly, studies from US and Europe are of little help in this is type of investigation and locally held health research is fundamental in determining future efficient steps.
